# The Diabetic Rapid Response Acute Foot Team: 7 Essential Skills for Targeted Limb Salvage

**Published:** 2009-05-05

**Authors:** Ryan H. Fitzgerald, Joseph L. Mills, Warren Joseph, David G. Armstrong

**Affiliations:** Southern Arizona Limb Salvage Alliance and College of Medicine, University of Arizona, Tucson

## Abstract

**Objective:** People with diabetes are prone to develop lower-extremity ulcerations and infections, both of which serve as major risk factors for limb amputation. The development of lower-extremity complications of diabetes is associated with increased morbidity and mortality. Recently, there has been increasing interest in the development of interdisciplinary teams to manage the myriad factors that complicate the treatment of high-risk patients, particularly in the perihospitalization period. **Methods:** This article presents 7 essential skills that necessarily allow the limb salvage team to appropriately manage the most common presenting comorbidities in patients with diabetes, including vasculopathy, infection, and deformity. **Results:** Seven essentials skills have been demonstrated to promote the greatest salvage outcomes, and these are the ability to (1) perform hemodynamic and anatomic vascular assessment with revascularization, as necessary; (2) perform neurologic workup; (3) perform site-appropriate culture technique; (4) perform wound assessment and staging/grading of infection and ischemia; (5) perform site-specific bedside and intraoperative incision and debridement; (6) initiate and modify culture-specific and patient-appropriate antibiotic therapy; and (7) perform appropriate postoperative monitoring to reduce risk of reulceration and infection. **Conclusions:** Utilization of these 7 essential skills as the core basis for interdisciplinary limb salvage team models will provide clinicians guidance when establishing such teams. Interdisciplinary teams have been demonstrated to improve quality and efficiency of patient care, thus improving overall outcomes and reducing amputation rates.

The incidence of diabetes mellitus worldwide has reached almost epidemic proportions, with nearly 26 million people affected by the disease in the United States alone.^1^,^2^ In concert with this increased incidence, there has been a significant rise in the observed comorbidities commonly associated with the disease process in patients with diabetes.^3^,^4^ Among these complications, lower-extremity manifestations are a significant source of patient comorbidity, mortality, and healthcare expense. It has been estimated that the lifetime risk of developing diabetic foot ulceration (DFU) is as high as 25% in patients with diabetes.^1^,^5^ In addition to the development of DFU, more than 50% of these ulcerations will become infected, accounting for nearly 20% of all diabetes-related hospital admissions, and therefore a significant portion of healthcare related costs—nearly $11 billion—in 2001.^6^

In patients with infected DFUs, underlying osteomyelitis is observed in as many as 65% of cases, and these infected ulcers constitute a major risk factor for nontraumatic lower-extremity amputation (LEA).^5^ Indeed, nearly 83% of all nontraumatic LEAs in the United States are secondary to complications associated with diabetes mellitus.^5^ It has been well documented that the consequences of major LEA in diabetic patients are severe, with an estimated 5-year postoperative survival rate of less than 50%, suggesting, in fact, that mortality associated with diabetic LEAs exceeds that of most cancers.^7^ It is therefore vital to provide early and effective diagnosis and management of patients with lower-extremity complications of diabetes in an effort to stem the current epidemic of limb loss.

Considering that the pathophysiology of lower-extremity limb loss in patients with diabetes is multifactorial and that vasculopathy and neuropathy are critical contributors, it is appropriate to utilize an interdisciplinary team approach to specifically address the varying factors that combine to create lower-extremity ulceration, infection, and subsequent amputation. Such interdisciplinary models have been demonstrated to be highly effective in reducing the incidence of nontraumatic amputations in the diabetic population. A search of the literature reveals that little has been published on the requisite skill sets of the interdisciplinary team to promote limb preservation in patients with diabetes. The authors have defined 7 vital abilities that a diabetic rapid response acute foot team (DRRAFT) should have in its armamentarium so that it might effectively manage the lower-extremity complications of diabetes.^8^

## THE TEAM

The diabetic rapid response acute foot team is an interdisciplinary team model whose core involves the ability to rapidly diagnose and provide effective treatment to patients with lower-extremity complications of diabetes, utilizing seven basic skill sets. The authors have previously advocated that the “irreducible minimum” regarding interdisciplinary units be oriented around treatment teams that are staffed by members of the vascular surgery and podiatric surgery specialties, with adjunctive team members being added as necessary via judicious use of consultation.^9^ The DRRAFT concept is the natural extension of this premise: Bringing the nuances from each individual specialty, the team collectively must possess the ability to perform 7 essential skills to be effective in promoting limb preservation. However, any clinician involved in the care of the diabetic patient with a passion for limb salvage can function as a member of the DRRAFT limb salvage team. Indeed, often times, geographic limitation will require that requisite team member roles be performed by physicians trained in global wound care, advanced practice nurses, or clinical nurse specialists.

## SEVEN ESSENTIAL SKILLS

The management of the lower-extremity manifestations of diabetes mellitus is a complex task, and it is necessary that practitioners involved in diabetic limb salvage address both the systemic and local factors that interact to generate significant comorbidity and mortality in this patient population. The major factors include vasculopathy and neuropathy, often in combination with foot deformity, and lead to the development of DFUs.^10^–^12^ The literature is clear that infected diabetic ulcerations present a major risk factor for LEA and therefore it is necessary to appropriately manage DFUs when they occur, including addressing the underlying etiology as well as dealing with an infection that may be present.^13^ Seven essential skills have been identified, and are detailed in the following text, which can be utilized in combination by DRRAFT members to effectively manage DFUs when they occur and prevent progression to LEA.

1. **The ability to perform hemodynamic and anatomic vascular assessment with revascularization, as necessary.**

Patients with diabetes often suffer from peripheral arterial disease with elements of microvascular and macrovascular diseases,^14^,^15^ although it is predominantly macrovascular disease that produces critical limb ischemia. Patients with critical limb ischemia are at significant risk for limb loss and require timely intervention to improve distal lower-extremity perfusion.^16^ Among patients with both vascular compromise and lower-extremity ulceration, it is necessary to address the patient's vascular status in order to promote effective wound healing and to reduce amputation risk.

It is vital, therefore, that DRRAFT members have readily at hand the capability to rapidly assess the lower-extremity circulation and potential for healing by noninvasive means. Although physical examination and handheld Doppler evaluation by an experienced clinician are extremely important, objective testing of lower-extremity perfusion is frequently required and can be accomplished by toe Doppler waveform analysis and toe pressure determinations, transcutaneous oxygen pressure (Tcpo_2_) measurements, and arterial duplex ultrasound. Depending on the results of these studies and the response of the index wound to care, further testing such as computed tomography angiography or magnetic resonance angiography may be indicated, although conventional arteriography is often preferred once a hemodynamic state insufficient to heal the foot has been documented, since a therapeutic procedure (angioplasty or stent placement) can be often performed at the same sitting as diagnostic arteriography.^17^–^19^ Early arteriography and rapid revascularization either by endovascular means or open surgical bypass are needed in patients with DFUs who have inadequate perfusion to limit persistent limb ischemia and subsequent tissue loss and to provide sufficiently increased distal flow to promote wound healing.^16^ Delays in the recognition and treatment of macrovascular occlusive disease compromise outcomes, delay wound healing, prolong hospital stays, and unnecessarily increase the risk of major limb amputation.^14^

2. **The ability to perform neurologic workup.**

Diabetic patients often develop neurologic symptoms as a consequence of long-standing hyperglycemia; these include motor, sensory, and autonomic neuropathy.^10^,^11^,^20^ These symptoms are involved at many levels in the development of lower-extremity ulcerations. Perhaps, most widely recognized of the neurologic symptoms common to diabetics is sensory neuropathy with loss of protective sensation (LOPS).^21^ These patients lose the “gift of pain.” In the absence of pain, diabetic patients are far more likely to develop ulcerations due to LOPS in the context of increased shear and pressure forces. In addition, motor neuropathy in the intrinsic musculature can lead to muscle imbalance, which creates foot deformity that, in conjunction with sensory neuropathy, can lead to the development of areas of increased forces that can progress to ulceration.

It is fundamental that DRRAFT members be able to appropriately evaluate the patient's neurologic status to establish LOPS via sensory neuropathy, as well as any concomitant elements of motor or autonomic neuropathy that can contribute to the development of lower-extremity ulceration.^1^ Loss of protective sensation can be evaluated via the 5.07 Semmes-Weinstein monofilament test, which is utilized to evaluate sensory perception at 10 locations along the dorsal and plantar aspects of the foot, and has been shown to demonstrate a 97% sensitivity and a 83% specificity in identifying patients at highest risk for diabetic foot ulcers.^22^ Motor neuropathy can be determined by evaluating muscular strength in the various muscle groups in the lower extremity. The diagnosis of neuropathy is important because it correlates with the risk of recurrence, dictates the intensity and type of follow-up that will be required, and mandates ongoing attention to prevention by regular assessment of footwear and attention to proper offloading.

3. **The ability to perform site-appropriate culture technique.**

When presented with infected lower-extremity ulcerations, the clinician must be able to reliably obtain useful culture data. The literature demonstrates that lower-extremity infections of diabetes are often polymicrobial, with an average of 2.25 pathogens per patient.^23^ Furthermore, results of superficial swab cultures taken from a wound are notoriously unreliable; one study demonstrated that superficial swabs of infected ulcerations identified deep soft-tissue pathogens in only 75% of cases.^24^ It is necessary that suitable deep culture specimens from a wound should be obtained to appropriately direct antibiotic therapy. *Staphylococcus aureus* and *Staphylococcus epidermidis*, *Enterococcus*, and *Streptococcus* species, particularly group B, β hemolytic, are most commonly observed; however, in this era of increasing drug-resistant organisms, methicillin-resistant *S. aureus* is being identified more and more frequently, especially in patients wih diabetes.^23^ In addiion, aerobic gram-negative rods (*Pseudomonas aeruginosa* being the most common) and obligate anaerobe species have also been observed in many cases, although their pathogeniciy is not clear. Lavery et al^25^ reported that only 36% of soft-tissue cultures yielded accurate bone pathogens; therefore, in those instances in which underlying osteomyeliis is suspected, bone biopsy is recommended in addiion to deep soft-tissue cultures to increase the likelihood of accurately identifying the appropriate causative microorganisms.^23^,^26^ Cultures of pathogens should be harvested before the patient receives any antibiotics, if at all possible, since this may affect the reliabiliy of the specimen.

4. **The abiliy to perform wound assessment and staging/grading of infection and ischemia.**

**Figure 1:**
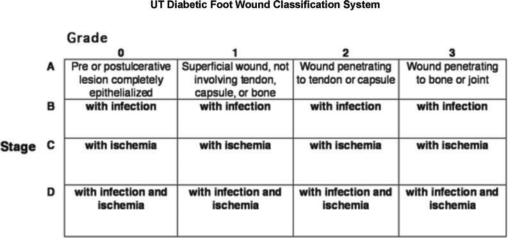
Universiy of Texas Diabetic Foot Wound Classification System.^27^

**Figure 2:**
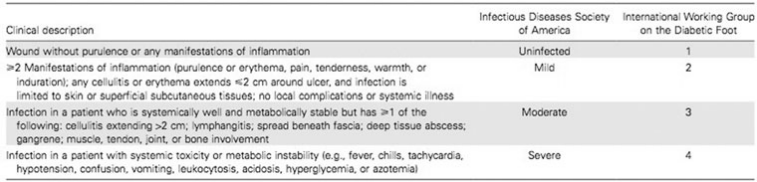
Infection classification systems.

**Figure 3:**
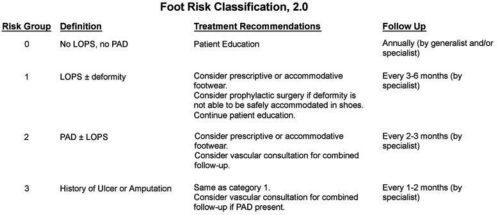
Diabetic foot risk classification system. LOPS indicates loss of protective sensation; PAD, peripheral arterial disease.^21^,^22^

When evaluating lower-extremiy ulcerations in the diabetic population, i is vial that practiioners be able to speak a lingua franca, or “common language,” regarding these wounds. Numerous classification systems have been proposed that seek to provide this common language; perhaps, the most universal is the Universiy of Texas Diabetic Foot Wound Classification System (Fig 1). This system provides a uniform basis for describing lower-extremiy diabetic ulceration wih regard to depth of wound and the presence (or absence) of ischemia and infection.^27^,^28^ Extending from grade 0 to grade 3, the depth of the wound is described, wih increasing depth of the wound noted as one moves along the horizontal axis. In this classification system, wounds are further staged according to the presence of infection, ischemia, or both. Utilization of validated classification systems, such as the Universiy of Texas Diabetic Foot Wound Classification System, can allow DRRAFT members to transcend the language of their particular specialties to effectively describe lower-extremiy diabetic wounds.^27^ While the Universiy of Texas system is useful for describing the ulceration iself, i may not be specific enough if infection is the primary presentation. Both the International Working Group on the Diabetic Foot and the Infectious Diseases Society of America Diabetic Foot Guidelines Commitee have independently developed very similar infection staging systems that are mostly interchangeable and have recently been statistically validated (Fig 2). These systems stage infection on the basis of severiy of infection presentation and from this clinical description suggest which the most common pathogenic organisms are and recommend empiric antibiotic therapy. American Diabetes Association (ADA) guidelines are included in number 7 in the following text, and Figure 3 represents ADA guidelines/classification.

5. **The abiliy to perform sie-specific bedside and intraoperative incision and debridement.**

Following appropriate assessment of vascular status and assessment of potential infection, i is necessary that DRRAFT members be able to provide timely incision and drainage to decompress areas of abscess formation as well as to provide appropriate debridement to remove all infected, nonviable, and necrotic soft tissue and bone. Such debridement limis the proximal spread of infection, obtains deep specimens for culture, and allows for tissue demarcation in the zones of tissue compromise.^11^

Numerous authors have advocated the use of debridement in the management of lower-extremiy infection, and i plays a vial role in diabetic limb salvage.^14^,^29^–^31^ Appropriate wound debridement is the starting point on the continuum of wound healing, and removal of necrotic, nonviable tissue down to a bleeding base is necessary to allow for wound bed preparation and the formation of healthy granular tissue.^32^ Furthermore, soft-tissue debridement is useful in stimulating quiescent cells observed in chronic wounds. Appropriate and timely tissue debridement is essential in turning the tide of infection and setting the patient down the path toward reconstructive efforts, wound healing, and functional limb preservation.^33^,^34^

6. **The abiliy to iniiate and modify culture-specific and patient-appropriate antibiotic therapy.**

To effectively control infection in the diabetic patient, i is vial that appropriate wound cultures be obtained.^23^ Patients wih almost all mild and some moderate infections can be treated wih oral antibiotics wih fairly specific activiy against aerobic gram-posiive organisms. Patients wih more severe infections should iniially be placed on empiric, broad antibiotic converge until more focused therapy can be iniiated on the basis of appropriate culture results.^23^,^31^ As previously discussed, the majoriy of the moderate to severe lower-extremiy infections in this patient population are polymicrobial, and considering the increasing rates of antibiotic-resistant strains of pathogens, i is vial that these patients receive appropriate antibiotic coverage. Toward this end, i is criical that DRRAFT members be able to effectively select appropriate empiric therapy and modify patient's antibiotic regimens in response to accurate culture and sensiiviy data.

7. **The abiliy to perform appropriate postoperative monioring to reduce risks of reulceration and infection.**

Just because iniial wound healing has been achieved does not guarantee that the battle is yet won. Diabetic patients wih previous ulceration are at significantly increased risk for reulceration.^35^,^36^ It is necessary that DRRAFT members have the abiliy to actively follow these high-risk patients throughout the post–wound healing phase. DRRAFT members should utilize the ADA Foot Risk Classification System, which quantifies patients into risk groups on the basis of vascular status, potential LOPS, as well as the presence or absence of deformiy, and then makes treatment and follow-up recommendations on the basis of increasing levels of risks (Fig 3).^10^,^37^ Patients wih history of ulceration or amputation are considered to be in risk group 3 and require stringent follow-up every 1 to 2 months for prophylactic evaluation. In addiion, the postulcerative patients should be encouraged to evaluate their lower-extremiy skin temperatures via dermal thermometer daily at home because studies have demonstrated that skin temperatures significantly increase before skin breakdown actually occurs.^38^ In this way, these high-risk patients can monior themselves and tirate their activiy accordingly.

## CONCLUSION

As the population ages and lifestyles change, the incidence and prevalence of diabetes mellius are increasing, and therefore i is incumbent upon clinicians involved in the care of patients wih diabetes to be adequately prepared to provide efficient, qualiy care to prevent lower-extremiy ulceration, infection, and ultimate amputation. The DRRAFT model proposes 7 essential skills that form a necessary core of the interdisciplinary limb salvage model. These skills provide for the rapid diagnosis and timely surgical management of diabetic patients wih lower extremiy compromise and should be the foundation upon which any interdisciplinary team be built.
